# Extraction and Yield Optimisation of Fucose, Glucans and Associated Antioxidant Activities from *Laminaria digitata* by Applying Response Surface Methodology to High Intensity Ultrasound-Assisted Extraction

**DOI:** 10.3390/md16080257

**Published:** 2018-07-30

**Authors:** Marco Garcia-Vaquero, Gaurav Rajauria, Brijesh Tiwari, Torres Sweeney, John O’Doherty

**Affiliations:** 1 School of Veterinary Medicine, Veterinary Sciences Centre, University College Dublin, Belfield, Dublin 4, Ireland; marco.garciavaquero@ucd.ie (M.G.-V.); torres.sweeney@ucd.ie (T.S.); 2 School of Agriculture and Food Science, University College Dublin, Lyons Research Farm, Celbridge, Co., Kildare W23 ENY2, Ireland; gaurav.rajauria@ucd.ie; 3 Department of Food Biosciences, TEAGASC, Food Research Centre, Ashtown, Dublin 15, KN3K, Ireland; brijesh.tiwari@teagasc.ie

**Keywords:** seaweed, fucoidan, laminarin, FRAP, DPPH, ultrasound-assisted extraction, innovative technology, optimization

## Abstract

The objectives of this study were to employ response surface methodology (RSM) to investigate and optimize the effect of ultrasound-assisted extraction (UAE) variables, temperature, time and amplitude on the yields of polysaccharides (fucose and total glucans) and antioxidant activities (ferric reducing antioxidant power (FRAP) and 1,1-diphenyl-2-picryl-hydrazyl radical scavenging activity (DPPH)) from *Laminaria digitata*, and to explore the suitability of applying the optimum UAE conditions for *L. digitata* to other brown macroalgae (*L. hyperborea* and *Ascophyllum nodosum*). The RSM with three-factor, four-level Box-Behnken Design (BBD) was used to study and optimize the extraction variables. A second order polynomial model fitted well to the experimental data with R^2^ values of 0.79, 0.66, 0.64, 0.73 for fucose, total glucans, FRAP and DPPH, respectively. The UAE parameters studied had a significant influence on the levels of fucose, FRAP and DPPH. The optimised UAE conditions (temperature = 76 °C, time = 10 min and amplitude = 100%) achieved yields of fucose (1060.7 ± 70.6 mg/100 g dried seaweed (ds)), total glucans (968.6 ± 13.3 mg/100 g ds), FRAP (8.7 ± 0.5 µM trolox/mg freeze-dried extract (fde)) and DPPH (11.0 ± 0.2%) in *L. digitata*. Polysaccharide rich extracts were also attained from *L. hyperborea* and *A. nodosum* with variable results when utilizing the optimum UAE conditions for *L. digitata*.

## 1. Introduction

Macroalgae are a diverse group of organisms capable of adapting to the extreme marine environmental conditions by producing multiple bioactive compounds. Marine macroalgae are considered a rich source of micro- and macronutrients with antioxidant activities, i.e., minerals, carotenoids, phenolic compounds, proteins and polysaccharides [1].

Macroalgal polysaccharides, particularly fucoidan and laminarin have a wide range of biological activities such as antioxidant, immunostimulatory and anti-microbial both in vitro and in vivo [2,3]. Fucoidans are a family of sulphated fucose-rich polysaccharides, built on a backbone of α-l-fucopyranose residues [4]. These polysaccharides are an integral part of the cell walls of brown macroalgae, playing a crucial role in the protection of seaweeds against environmental challenges [5]. Laminarins are glucan-polysaccharides containing 1,3-linked β-d-glucose residues with different degrees of 6-O branching and β-(1,6) intrachain links [6]. Hence, glucans are polysaccharides of d-glucose monomers that act as energy reserve compounds stored in vacuoles inside the macroalgal cells [7].

The traditional extraction methods employed for polysaccharides involve the use of one or several solvents used alone or in combination with high temperatures [2]. Innovative technologies are currently being explored to generate novel extraction protocols, aiming to obtain higher yields of bioactive compounds and to develop more environmentally friendly processes, with lower energy consumption, time and cost of extraction [8]. Ultrasound-assisted extraction (UAE) is one of the most promising innovative technologies used to date [9]. The enhancement in the extraction of bioactive compounds achieved by UAE is mainly attributed to the effect of cavitations in the solvent [10]. The process of cavitation involves nucleation, growth and collapse of bubbles in a liquid, driven by the bulk pressure variation created by the passage of the ultrasound waves. The cavitation creates physical effects, such as velocity/pressure shockwaves that causes cell disruption, and micro-turbulences that mixes and accelerates the extraction of bioactive compounds through the cell membranes [8,10]. The UAE has been utilized to extract multiple functional molecules from plants [11,12,13], macroalgae [14,15] and microalgae [16].

Recent research has indicated the need for further scientific efforts to study in detail these novel extraction processes of bioactive compounds, in order to obtain consistent protocols accounting for the biodiversity of polysaccharides in different macroalgal species [17]. The optimization of the innovative extraction technologies should be performed focusing on the yields of bioactive compounds and on the biological activities of the molecules obtained [17]. Novel optimization techniques, such as response surface methodology (RSM), which is a multivariate statistic approach, have recently displaced other traditional optimization strategies (i.e., “one-variable-at-a-time”) [2]. The RSM is based on the fit of a polynomial equation to the experimental data with the objective of making statistical provisions. The advantages of RSM over traditional strategies include the possibility to include in the model the interactive effects among the variables studied, while being less time and cost-consuming by reducing the number of experiments needed in the process of optimization [2].

Few studies have described the use of UAE to obtain fucoidan and laminarin (estimated as fucose and total glucans) from macroalgae. Most studies using macroalgae focused on obtaining high yields of total carbohydrates [18], or on analysing glucans and/or fucose without optimizing the technologies used [7,15,19] or on optimizing few extraction variables without including the biological activity of the compounds in the statistical models [20]. For instance, in a previous study conducted by Kadam et al. [20], extraction parameters such as time and amplitude were optimized to enhance the yield of bioactive compounds (total phenolics, fucose and uronic acid) from *A. nodosum* while temperature parameter was neglected during the optimization. In another study, the same authors investigated the efficiency of ultrasound assisted extraction of laminarin only and compared it to the traditional solid-liquid extraction methods without optimizing any UAE parameters. Furthermore, the chemical structure of laminarin varies depending on the variety and origin of seaweed. For instance, β-glucans derived from *L. digitata* contain small levels of β-(1-6)-linked side-chains while β-glucans derived from *L. hyperborea* only contain linear β-(1-3)-linked residues [21]. Therefore, the objectives of this study were to employ response surface methodology to (1) investigate the effect of UAE variables (extraction temperature, time and ultrasonic amplitude) on the yields of bioactive polysaccharides (fucose and total glucans) and the antioxidant activities (FRAP and DPPH) of extracts from *L. digitata*, and to (2) optimize the UAE variables to obtain high yields of polysaccharides and optimum antioxidant activities from *L. digitata* and other economically important brown macroalgae (*L. hyperborea* and *A. nodosum*).

## 2. Results and Discussion

### 2.1. Modelling the Extraction of Polysaccharides and Antioxidant Activity

The matrix design and the experimental responses (fucose, total glucans, FRAP and DPPH) for each run are presented in Table 1. There was considerable variation in the results obtained across the different parameters with ranges for: fucose (900.6 to 1257.7 mg/100 g ds), total glucans (774.9 to 1014.4 mg/100 g ds), FRAP (6.6 to 15.1 µM trolox/mg fde) and DPPH (9.8 to 15.3%). The highest yields of fucose (1257.7 mg/100 g ds) were obtained at an ultrasonic amplitude of 70%, at 80 °C for 30 min, while total glucans were maximum (1014.4 mg/100 g ds) using higher amplitudes (100%) and lower temperature and time during UAE (60 °C for 10 min). The antioxidant activities of FRAP (15.28 µM trolox/mg fde) and DPPH (15.10%) were optimum using milder UAE conditions (40% ultrasonic amplitude at 40 °C for 20 min).

A second order polynomial model fitted well to the experimental data (Table 2) with low standard error and regression co-efficient (R^2^) values of 0.79, 0.66, 0.64, 0.73 for fucose, total glucans, FRAP and DPPH, respectively. The ANOVA for the response surfaces of Table 2 identified that the linear models were significant for fucose (*p* < 0.05) and showed a tendency towards significance for FRAP response (*p* < 0.1). The quadratic model identified a tendency towards significance for DPPH (*p* < 0.1) and the interactions or cross-products among the extraction parameters studied were non-significant. Thus, only the linear and the quadratic effects of the independent factors were significant on the response surfaces in the current experimental design.

The significance of the three experimental variables affecting the extraction of polysaccharides and antioxidant activity of extracts generated from *L. digitata* can be determined from the model coefficients, multiple determinations and probabilities generated from the response surface regression (RSREG) and evaluated using ANOVA analysis (Table 3). The regression coefficients provided in Table 3 indicate the effect of every parameter on the experimental responses; the magnitude of the coefficients is related to the weight of its effect and the positive and negative signs indicate an increase and decrease in the experimental responses, respectively. The time (β2) and amplitude (β3) of extraction significantly affected (*p* < 0.05) and tended to influence (*p* < 0.1), respectively, the antioxidant power of the seaweed extracts measured as DPPH. The quadratic effect of amplitude (β33) significantly influenced (*p* < 0.05) the DPPH radical scavenging activities of the extracts, while the temperature (β11) tended to (*p* < 0.1) influence the levels of fucose. No significant interactions or cross-products were appreciated for any experimental response, with tendencies to significance (*p* < 0.1) in the case of total glucans (time and amplitude, β23) and DPPH (temperature and time, β12).

Equations (1)–(4) describe the influence of temperature (X_1_), time (X_2_) and ultrasonic amplitude (X_3_) on the extraction of fucose, total glucans, FRAP and DPPH of extracts from *L. digitata*:

Fucose (mg/100 g ds) = 1277 − 13.1 X_1_ − 9.2 X_2_ − 0.37 X_3_ + 0.1961 X_1_ X_1_ + 0.024 X_2_ X_2_ + 0.0418 X_3_ X_3_ + 0.092 X_1_ X_2_ − 0.0961 X_1_ X_3_ + 0.070 X_2_ X_3_ (1)

Total glucans (mg/100 g ds) = 591 + 8.1 X_1_ − 2.0 X_2_ − 0.21 X_3_ − 0.0666 X_1_ X_1_ + 0.497 X_2_ X_2_ + 0.0402 X_3_ X_3_ − 0.097 X_1_ X_2_ − 0.0009 X_1_ X_3_ − 0.228 X_2_ X_3_ (2)

FRAP (µM trolox/mg fde) = 14 + 0.079 X_1_ − 0.145 X_2_ + 0.044 X_3_ − 0.00067 X_1_ X_1_ + 0.00428 X_2_ X_2_ − 0.000164 X_3_ X_3_ − 0.00103 X_1_ X_2_ − 0.00057 X_1_ X_3_ − 0.00055 X_2_ X_3_ (3)

DPPH (%) = 1.1 + 0.489 X_1_ + 1.383 X_2_ − 0.444 X_3_ − 0.00255 X_1_ X_1_ − 0.01718 X_2_ X_2_ + 0.00304 X_3_ X_3_ − 0.01076 X_1_ X_2_ + 0.00022 X_1_ X_3_ − 0.00124 X_2_ X_3_ (4)

**Figure 1:**
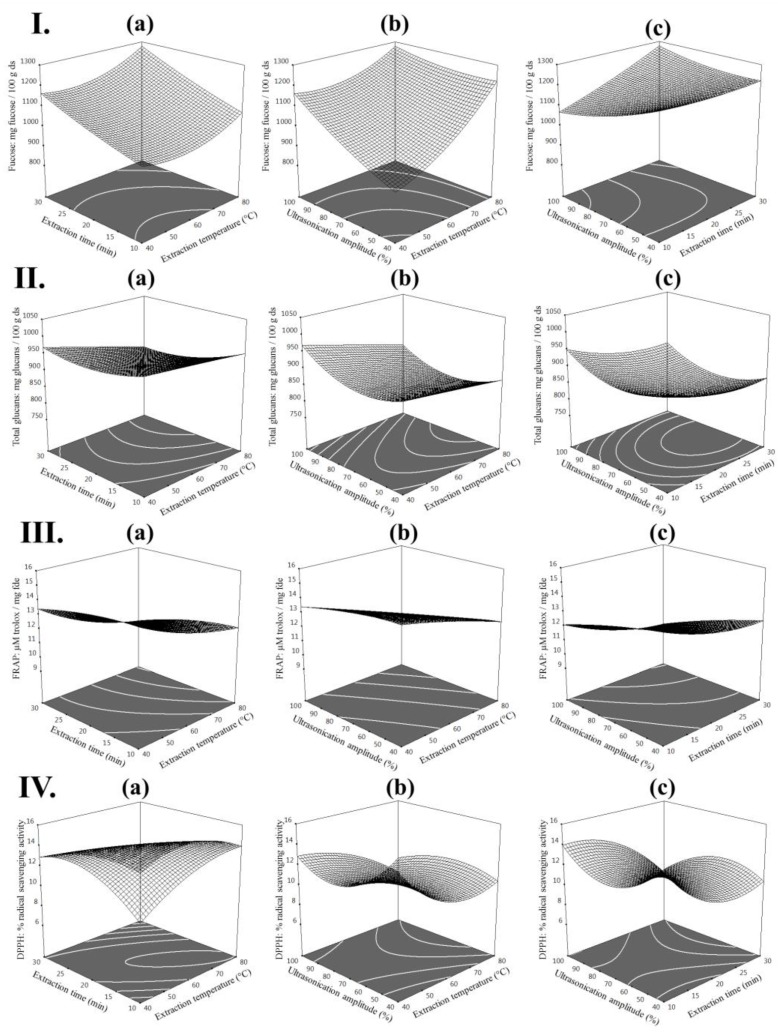
Contour plots (2D) and response surface plots (3D) of (**I**) fucose (mg/100 g dried seaweed (ds)); (**II**) total glucans (mg/100 g ds); (**III**) FRAP (µM trolox/mg freeze-dried extract (fde)) and (**IV**) DPPH (%) extracted from *Laminaria digitata* as a function of (**a**) time to temperature (amplitude = 100%) (**b**) temperature to amplitude (time = 30 min) and (**c**) amplitude to time (temperature = 80 °C).

Furthermore, contour plots (2D) and response surface plots (3D) were generated from the model equations to visualize the relationship between the UAE variables of extraction and the yields of fucose, total glucans and the antioxidant activities (FRAP and DPPH) of extracts from *L. digitata* (see Figure 1). These figures provided a visual interpretation of the mutual interactions between the 3 extraction variables and the expected responses (fucose, total glucans, FRAP and DPPH). Each graphic represents the effect of 2 extraction variables on the experimental response when the non-represented extraction variable is kept at its maximum; thus, these figures are a useful tool to predict the optimum extraction conditions and predicted values of polysaccharides and their related antioxidant activities.

### 2.2. Optimization of the Extraction of Polysaccharides and Antioxidant Activity

The current study focuses on the extraction of both fucose and glucans together along with their antioxidant activity by optimizing time, temperature and amplitude. All extraction parameters were optimized by using a more powerful semi-industrial ultra-sonication device (power 500 W, 20 kHz), compared to a lab grade ultra-sonication device used in previous studies [7,20]. Optimum conditions were determined aiming to maximize the yields of (i) fucose (condition 1), (ii) total glucans (condition 2), (iii) antioxidant activities (FRAP and DPPH) (condition 3) and (iv) yield of polysaccharides and antioxidant activities combined (condition 4). The levels of the three independent parameters used in UAE (temperature, time and ultrasonication amplitude), together with the predicted values and the experimental results obtained from *L. digitata* extracts are summarized in Table 4. The predicted values of the theoretical model for the four optimum conditions described were confirmed with the experimental data with the exception of the FRAP values, which were lower than the predicted values in both conditions 3 and 4.

The optimum UAE extraction conditions to obtain high yields of fucose from *L. digitata* were temperature (80 °C), time (30 min) and ultrasonication amplitude (40%; condition 1; Table 4). There is some conflicting data in the literature with regard to the influence of these conditions on the yields of fucose. Previous studies using UAE did not identify an influence of time or amplitude on the fucose content of extracts from *A. nodosum* [20]. Our results suggested that temperature is a critical factor for getting higher yield of both fucose and glucans along with total antioxidant activity, which was neglected in previous studies [7,20]. Our results are in agreement with Ale et al. [22] wherein the temperature and time of extraction also had an influence on the extraction of fucose from *Sargassum* spp. using conventional extraction techniques, with optimum extraction conditions obtained at temperatures of 90 °C over a 4 h period. However, previous researchers optimizing UAE conditions to obtain bioactive compounds from plants identified an influence of temperature, time and various ultrasonication parameters (i.e., frequency and power) on the yields of polysaccharides [23,24]. 

The optimum UAE extraction conditions to obtain high yields of total glucans from *L. digitata* were temperature (52.5 °C), time (10 min) and ultrasonication amplitude (100%; condition 2; Table 4). High ultrasonication amplitudes were also required to recover glucans from mushroom by-products (*Agaricus bisporus*) with the highest yields of glucans obtained applying high ultrasonic amplitudes (100 μm) for 15 min, followed by 1 h of precipitation with ethanol [25]. A previous study carried out by Kadam et al. [7] using 0.1 M HCl showed an increased extraction of glucans from *L. hyperborea* and *A. nodosum* at 60% of ultrasonic amplitude for 15 min, although the optimization of the UAE parameters was not performed [7].

The mild extraction conditions needed to preserve the antioxidant activities (FRAP and DPPH) of extracts from *L. digitata* (temperature 40 °C, time 30 min and amplitude 40%; condition 3; Table 4) could be due to the antioxidant power of other thermolabile compounds that could be present in the crude extracts, such as proteins/peptides [1] and polyphenols [26,27]. In fact, previous studies optimizing UAE to achieve phenolic compounds from brown macroalgae (*Hormosira banksia*) obtained maximum phenolic contents using low temperatures (30 °C) at medium sonication power (60%) for 60 min [28].

The optimum conditions to obtain both high yields of polysaccharides and antioxidant activities were of temperature (76 °C), time (10 min) and ultrasonication amplitude (100%; condition 4; Table 4). To our knowledge there are no studies presented in the literature that aim to optimize the yields of polysaccharides and its antioxidant activities from any species of seaweed.

### 2.3. Application of Optimal UAE Conditions in other Brown Macroalgae

The applicability of the four optimum conditions for *L. digitata* was subsequently explored to generate polysaccharide rich extracts from other brown macroalgae with commercial value (*L. hyperborea* and *A. nodosum*). The contents of fucose, total glucans and antioxidant activities (FRAP and DPPH) of extracts from *L. hyperborea* and *A. nodosum* using optimal UAE conditions are compiled in Table 5. *L. hyperborea* extracts had higher contents of total glucans and DPPH activities, being approximately 10 and 4 fold higher than the values obtained from *L. digitata*, respectively. *A. nodosum* extracts showed powerful antioxidant activities (FRAP and DPPH) when compared to both *Laminaria* species. Previous studies aiming the UAE of fucose and glucans from brown macroalgae achieved extracts containing 87.06 mg fucose/g from *A. nodosum* [20] and 5.29–6.24 mg glucans/100 mg from *L. hyperborea* and *A. nodosum*, although the antioxidant activity of these extracts was not reported [7].

There is substantial variation in the concentration of the various different bioactive compounds extracted from the different macroalgal species in this study. Previous studies reported variable contents of polysaccharides in brown macroalgae depending on the seaweed species and season of collection [29,30]. Low fucoidan levels were described in *A. nodosum* collected in March compared to *Fucus serratus* and *F. vesiculosus* collected in the same season [29], while high concentration of glucans were reported in *L. hyperborea* compared to *L. digitata*, *Saccharina latissima* and *Alaria esculenta*, with maximum levels of these polysaccharides in all macroalgal species described during the summer and autumn [30]. It is important to note that the macroalgae utilised in this study were all collected at the same time and from the same location; thus, the differences in the macroalgal extracts could be also attributed to inter-species variations in polysaccharide contents in the raw biomass.

## 3. Materials and Methods

### 3.1. Macroalgal Biomass

Brown macroalgae (*L. digitata*, *L. hyperborea* and *A. nodosum*) were harvested in November 2016 by Quality Sea Veg Ltd., Co., Donegal, Ireland. Samples were cleaned of epitopes and oven-dried at 50 °C for 9 days. Dried seaweed samples were milled to a 1 mm particle size using a Christy and Norris Hammer Mill (Chelmsford, UK). Samples were vacuum-packed and stored at room temperature for further analysis.

### 3.2. Chemicals

l-(-)-Fucose, ascorbic acid, citric acid, sodium acetate, ferric chloride, sodium phosphate dibasic, methanol, triton™ X-100, sulfuric acid (95–97%), l-cysteine, potassium hydroxide, hydrochloric acid, 1,1-diphenyl-2-picryl-hydrazyl (DPPH), 2,4,6-tripyridyl-s-triazine (TPTZ), 6-hydroxy-2,5,7,8-tetramethylchromane-2-carboxylic acid (trolox) were purchased from SIGMA (Sigma-Aldrich, Saint Louis, MO, USA). Acetic acid and sodium hydroxide were purchased from VWR (VWR International, Radnor, PA, USA). Enzymatic glucan assay kits K-YBGL were purchased from Megaenzyme (Megazyme International Ltd., Bray, Ireland). Distilled water was used in all the extraction and analytical procedures.

### 3.3. Ultrasound-Assisted Extraction (UAE)

**Figure 2:**
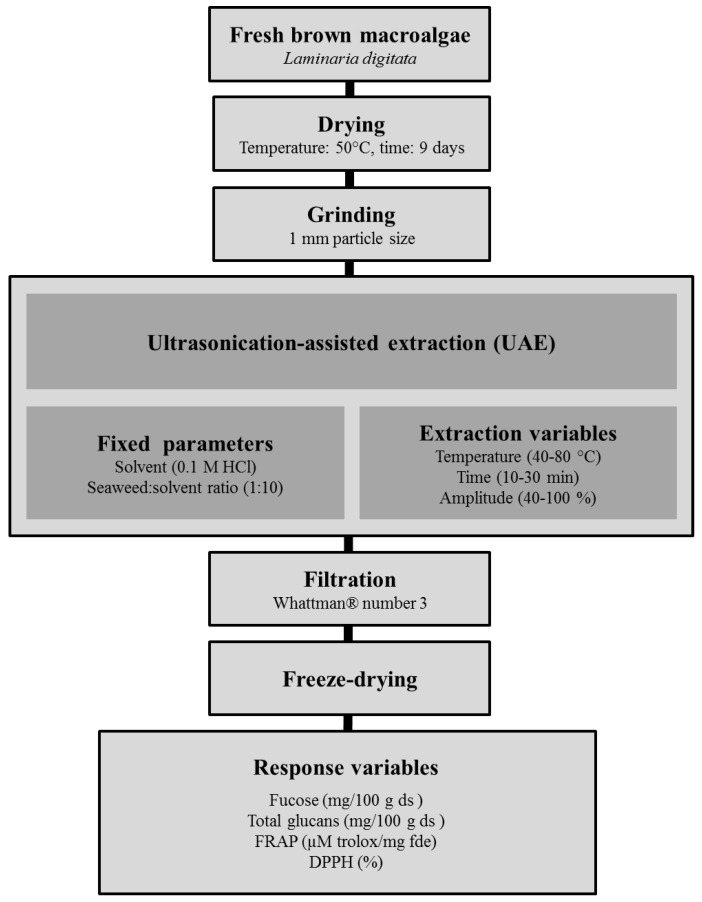
Scheme summarizing the pre-treatments of the fresh macroalgae and the ultrasound-assisted extraction (UAE) used to generate seaweed extracts.

The pre-treatment of the seaweed samples and the UAE process performed in this study is presented schematically in Figure 2. 10 g of *L. digitata* powder was mixed with 0.1 M HCl (1:10, *w*/*v*) for 10 min before starting the extraction procedures. The selection of 0.1 M HCl as extraction solvent and the seaweed:solvent ratio (1:10) was based on previous studies on the effect of both parameters on the yield of polysaccharides from various macroalgae [4].

Ultrasound-assisted extraction (UAE) of the samples was performed using semi-industrial grade UIP500hdT ultrasonic processor (maximum nominal power 500 W, 20 kHz, Hielscher Ultrasound technology, Teltow, Germany). The extraction variables temperature (°C), time (min) and amplitude (%) were adjusted according to the matrix design described in detail in Section 3.5. Each extraction condition was performed in duplicate, the seaweed residues were filtrated through Whattman^®^ number 3 (GE Healthcare, Buckinghamshire, UK) and the supernatants combined. The combined extracts were freeze-dried in an industrial scale freeze-drier (FD80 model 119, Cuddon Engineering, Blenheim, New Zealand), vacuum sealed and stored at −20 °C until further analysis.

### 3.4. Composition of the Macroalgal Extracts

All composition analyses were performed in duplicate. The fucose and total glucan concentrations of the macroalgal extracts were analysed together with their antioxidant activity (ferric reducing antioxidant power (FRAP) and 1,1-diphenyl-2-picryl-hydrazyl (DPPH)) as described in the following sections:

#### 3.4.1. Fucose Determination

Fucoidan contents were estimated performing fucose measurements as described by Dische and Shettles [31] with slight modifications. Briefly, 1 mL of fucose standards (ranging from 0.005 to 0.1 mg/mL) and macroalgal extracts at appropriate dilutions were added to 4.5 mL of a mixture 1:6 of water:sulfuric acid. The mixtures were warmed for 10 min at 22 °C followed by placing the samples 10 min at 100 °C in a water bath. The samples and standards were cooled at room temperature, 0.1 mL of 3% cysteine hydrochloride were added and stored for 60 min at room temperature. The absorbance of the standards and extracts were read at 396 (A_396_) and 430 nm (A_430_) in a microplate reader (Epoch, BioTek, Winooski, VT, USA). The fucose content of the samples was determined against the fucose standard at effective absorbance of A_396_–A_430_. The fucose values were expressed as mg fucose per 100 g dried seaweed (ds).

#### 3.4.2. Total Glucan Determination

The total glucan contents of the macroalgal extracts were determined enzymatically using the enzymatic kit K-YBGL (Megaenzyme International Ltd., Bray, Ireland) according to the manufacturer’s instructions. Briefly, 100 mg of dried and milled samples and positive control (yeast β-glucan) were weighed and mixed with 1.5 mL of concentrated HCl (37% *w*/*v*). The samples were mixed and warmed at 30 °C for 45 min followed by the addition of 10 mL of distilled water and incubation in a shaking water bath (100 °C, 100 rpm and 2 h). Samples were cooled at room temperature, neutralized with 2 M KOH and adjusted to 100 mL with sodium acetate buffer (pH 5.0). Samples were centrifuged at 1500 g during 10 min and the supernatants collected. Duplicate subsamples of each supernatant (0.1 mL) were mixed thoroughly with 0.1 mL of a solution containing exo-1,3-β-glucanase (20 units (U)/mL) and β-glucosidase (4 U/mL) and incubated in a water bath at 40 °C for 60 min. Each subsample, together with blanks (0.2 mL sodium acetate buffer pH 5.0) and glucose standards (0.1 mL of glucose standard (1 mg/mL) and 0.1 mL of acetate buffer pH 5.0), were incubated with 3 mL of glucose-oxidase-peroxidase-reagent (GOPOD) at 40 °C for 20 min. The absorbance of glucose standards and subsamples were read at 510 nm against reagent blank (UVmini-1240, Shimadzu, Kyoto, Japan). Total glucans were calculated using Mega-Calc™ provided by Megazyme (Megaenzyme International Ltd., Bray, Ireland). The total glucan values were expressed as mg total glucans per 100 g ds.

#### 3.4.3. Antioxidant Activity

##### Ferric Reducing Antioxidant Power (FRAP)

The ferric reducing ability related antioxidant potential of the extracts was studied using the FRAP method described by Benzie and Strain [32] modified by Bolanos de la Torre, et al. [33]. Solutions containing the extracts (1 mg/mL) were prepared in Milli Q water. Trolox at concentrations ranging from 15–420 µM were used as standard. The FRAP working solution was freshly prepared by mixing 10:1:1:1.4 of acetate buffer (300 mM, pH 3.6), ferric chloride (20 mM in Milli Q water), 2,4,6-Tripyridyl-s-Triazine (TPTZ) (10 mM in 40 mM HCl) and Milli Q water, respectively. The reaction was initiated in a Greiner CELLSTAR^®^ 96 flat bottom microplate by adding 280 µL of FRAP working solution to 20 µL of the test compound (extracts at 1 mg/mL) or standard. The samples were incubated at 37 °C in the dark for 30 min and the absorbances were read at 593 nm. The FRAP values were expressed as µM trolox equivalents per mg (freeze-dried seaweed extract) fde.

##### 1,1-Diphenyl-2-Picryl-Hydrazyl (DPPH) Radical Scavenging Activity

The DPPH inhibition assay was performed according to the method described by Nicklisch and Waite [34] with slight modifications. Briefly, freeze-dried macroalgal extracts and positive control (ascorbic acid) were dissolved at a concentration of 1 mg/mL in 0.1 M citrate phosphate buffer pH = 5 with 0.3% (*v*/*v*) Triton X-100. The initial absorbance values of the tested samples, positive control and blank solutions (190 µL) were read in a Greiner CELLSTAR^®^ 96 flat bottom in a microplate reader (Epoch, BioTek, Winooski, VT, USA). The reaction was started by adding to each well 10 µL of a 2 mM solution of DPPH in methanol to give a final DPPH concentration of 100 µM in each well. The plates were incubated in the dark at room temperature for 30 min and the final absorbance of the reaction was read at 515 nm. The initial absorbance readings were subtracted from the final readings and the% radical scavenging activities were calculated using the following equation:

% DPPH inhibition = ((Abs Blank − Abs Inhibitor)/Abs Blank) × 100(5)

where Abs Blank is the absorbance of the DPPH solution without any test compounds and the Abs Inhibitor is the absorbance of the tested samples or positive control after the reaction takes place.

### 3.5. Experimental Design and Statistical Analysis

The optimization of the extraction of bioactive compounds from *L. digitata* was performed using RSM. A Box-Behnken Design with 3 independent variables, each at 4 levels, was employed in this study, requiring a total of 17 experiments for the optimization of the UAE variables. The experimental order was randomized and the levels of the independent variables temperature (40–80 °C), time (10–30 min) and ultrasonic amplitude (40–100%) were coded and listed with the original values in Table 6. The experimental design matrix and the extraction yields of fucose (mg/100 g ds), total glucans (mg/100 g ds), FRAP (µM trolox/mg fde) and DPPH (%) are compiled in Table 1. The results were analysed using response surface regression (RSREG) (SAS version 9.2) fitted to the following second-order polynomial model:

(6) $$Y=\beta 0+\sum_{i=1}^{3}\beta_{i}X_{i}\sum_{i=1}^{3}\beta_{ii}X_{i}^{2}+\sum_{i}^{3-1}\sum_{j}^{3}\beta_{ij}X_{i}X_{j}$$

where, *Y* is the predicted response (fucose, total glucans, FRAP and DPPH); *β*0 is the constant coefficient; *β_i_* is the linear coefficient; *β_ii_* is the quadratic coefficient; *β_ij_* is the cross product coefficients; *X_i_* and *X_j_* are independent variables. Plots combining contour (2D) and response surface (3D) were generated using Design Expert (v.11) software. The plots show the variation in the responses obtained from multiple combinations of 2 independent variables while holding one of the components constant in the second-order polynomial model. The validity of the model was determined by comparing the experimental and predicted values.

## 4. Conclusions

Ultrasound-assisted extraction (UAE) was studied for the extraction of polysaccharides (fucose and glucans) and antioxidant activities (FRAP and DPPH) from *L. digitata*. Response surface methodology was employed to investigate the effect of the UAE variables (temperature, time and ultrasonic amplitude) on the macroalgal extracts to enhance the yields of polysaccharides and its antioxidant activities. The UAE parameters studied showed significant influence on the levels of fucose, FRAP and DPPH. Levels of 1060.75 mg/100 g ds, 968.57 mg/100 g ds, 8.70 µM trolox/mg fde and 11.02% were obtained for fucose, total glucans, FRAP and DPPH respectively at optimized conditions of temperature (76 °C), time (10 min) and ultrasonic amplitude (100%) using 0.1 M HCl as solvent. The UAE conditions described were then applied successfully to other economically relevant brown macroalgae (*L. hyperborea* and *A. nodosum*) to obtain polysaccharide rich extracts. This study demonstrates the applicability of UAE to enhance the extraction of bioactive polysaccharides from various macroalgal species.

## Figures and Tables

**Table 1:** Matrix design and experimental responses expressed as mean ± standard deviation of the mean (*n* = 6).

Run Order	Extraction Variables			Experimental Responses ^γ^			
	Temperature (°C)	Time (min)	Amplitude (%)	Fucose (mg/100 g ds)	Total Glucans (mg/100 g ds)	FRAP (µM Trolox/mg fde)	DPPH (%)
1	80	30	70	1257.7 ± 9.7	866.5 ± 64.0	11.9 ± 0.5	6.6 ± 1.5
2	60	20	70	1108.3 ± 16.0	858.5 ± 60.1	14.9 ± 0.9	9.8 ± 0.7
3	40	10	70	904.5 ± 13.6	864.1 ± 49.9	14.6 ± 0.5	8.0 ± 1.7
4	60	20	70	908.0 ± 12.6	842.9 ± 55.3	11.2 ± 0.2	10.8 ± 2.8
5	40	20	40	900.6 ± 28.5	927.3 ± 57.4	15.3 ± 1.1	15.1 ± 1.4
6	60	20	70	995.4 ± 23.8	887.5 ± 64.5	12.3 ± 0.3	14.8 ± 1.9
7	40	20	100	1112.9 ± 14.8	942.2 ± 53.4	14.2 ± 1.0	14.0 ± 1.0
8	60	10	40	1033.6 ± 16.5	917.6 ± 65.5	14.7 ± 0.7	14.3 ± 1.4
9	40	30	70	965.1 ± 14.6	903.3 ± 52.4	13.5 ± 0.7	10.9 ± 1.0
10	60	10	100	959.1 ± 35.3	1014.4 ± 37.0	13.9 ± 0.9	12.3 ± 1.3
11	60	30	100	1151.6 ± 13.7	928.6 ± 100.8	12.1 ± 0.3	11.4 ± 0.8
12	60	20	70	998.5 ± 16.4	921.0 ± 48.2	13.7 ± 0.5	10.7 ± 1.9
13	80	20	40	1170.2 ± 21.4	882.2 ± 98.2	12.3 ± 0.2	13.5 ± 1.6
14	80	20	100	1147.3 ± 9.3	857.6 ± 74.1	9.8 ± 0.4	13.0 ± 1.0
15	60	20	70	1021.7 ± 21.1	774. 9 ± 71.2	14.4 ± 0.8	14.8 ± 1.1
16	60	30	40	1010.7 ± 3.2	841.2 ± 38.1	13.6 ± 0.2	14.9 ± 0.8
17	80	10	70	1161.5 ± 19.4	881.1 ± 49.4	13.8 ± 0.5	12.3 ± 1.5

**^γ^** The units of the experimental responses are expressed as follows: fucose (mg/100 g dried seaweed), total glucans (mg/100 g dried seaweed), FRAP (µM trolox/mg freeze-dried seaweed extract) and DPPH (% radical scavenging activity).

**Table 2:** Analysis of variance describing the effect of the treatment variables on the response variables (fucose, total glucans, FRAP and DPPH) as linear, quadratic and interactive terms.

Coefficient	Response Variables			
	Fucose	Total Glucans	FRAP	DPPH
Linear	6.01 *	1.95	3.74 ^a^	0.72
Quadratic	1.82	1.29	0.2	3.98 ^a^
Cross product	0.89	1.39	0.13	1.66
Lack of fit (p)	0.61	0.39	0.73	0.89
Total model	2.91 ^a^	1.55	1.36	2.12
RSME	78.42	69.95	1.36	1.96
CV	7.39	9.11	10.21	16.11
R^2^	0.79	0.67	0.64	0.73

* Significant at *p* < 0.05. ^a^ Tendency towards significance at *p* < 0.1.

**Table 3:** Regression coefficients and ANOVA of regression parameters of the predicted response surface quadratic models.

Coefficients ^Ϸ^	Response Variables ^γ^			
	Fucose	Total Glucans	FRAP	DPPH
β0	1276.83 ^a^	591.22	14.28	1.13
	(570.50)	(506.71)	(9.88)	(14.27)
Linear				
β1	−13.11	8.06	0.08	0.49
	(13.03)	(11.57)	(0.23)	(0.33)
β2	−9.19	−1.98	−0.14	1.38 *
	(21.53)	(19.12)	(0.71)	(0.54)
β3	−0.37	−0.21	0.04	−0.44 ^a^
	(7.64)	(6.79)	(0.75)	(0.19)
Quadratic				
β11	0.20 ^a^	−0.067	−0.0006	−0.002
	(0.09)	(0.085)	(0.002)	(0.002)
β22	0.024	0.5	0.004	−0.02
	(0.38)	(0.34)	(0.007)	(0.01)
β33	0.042	0.04	−0.0002	0.003 *
	(0.04)	(0.038)	(0.0007)	(0.001)
Cross product				
β12	0.092	−0.097	−0.001	−0.01 ^a^
	(0.2)	(0.17)	(0.004)	(0.005)
β23	0.07	−0.23 ^a^	−0.0005	−0.001
	(0.13)	(0.12)	(0.002)	(0.003)
β13	−0.1	−0.001	−0.0006	0.0002
	(0.065)	(0.058)	(0.001)	(0.002)

^γ^ The units of the experimental responses are expressed as follows: fucose (mg/100 g dried seaweed), total glucans (mg/100 g dried seaweed), FRAP (µM trolox/mg freeze-dried seaweed extract) and DPPH (% radical scavenging activity). The standard errors of the estimated coefficients are presented in parentheses. ^Ϸ^ Estimated coefficients of the model are: β0 (constant coefficient), linear regression coefficients (β1, β2 and β3), quadratic (β11, β22 and β33) and interaction (β12, β23, β13) effects of the model referred to the variables X1 (temperature), X2 (time) and X3 (amplitude). * Significant at *p* < 0.05. ^a^ Tendency towards significance at *p* < 0.1.

**Table 4:** Optimum conditions, predicted values and experimental responses of fucose, total glucans and antioxidant activities (FRAP and DPPH) from *Laminaria digitata*.

Optimum Conditions	Targeted Compounds-Bioactivities ^γ^	Parameters of Extraction			Predicted Values (95% CI) ^a^	Experimental Response (Mean ± SEM) ^b^
		Temperature (°C)	Time (min)	Amplitude (%)		
Condition 1	Fucose	80	30	40	Fucose (1061.5–1494.4)	Fucose (1147.6 ± 8.4)
Condition 2	Total glucans	52.5	10	100	Total glucans (809.6–1105.5)	Total glucans (1065.6 ± 5.8)
Condition 3	FRAP DPPH	40	30	40	FRAP (10.8–18.3) DPPH (10.3–21.1)	FRAP (10.3 ± 0.8) DPPH (11.5 ± 0.8)
Condition 4	Fucose Total glucans FRAP DPPH	76	10	100	Fucose (922.2–1312.5) Total glucans (746.5–1093.2) FRAP (9.1–15.8) DPPH (9.1–18.8)	Fucose (1060.7 ± 70.6) Total glucans (968.6 ± 13.3) FRAP (8.7 ± 0.5) DPPH (11.0 ± 0.2)

^a^ The predicted values were expressed as 95% confidence intervals. ^b^ Experimental responses were expressed as mean ± standard deviation of the mean. Number of readings (*n* = 6). ^γ^ The units of the experimental responses are expressed as follows: fucose (mg/100 g dried seaweed), total glucans (mg/100 g dried seaweed), FRAP (µM trolox/mg freeze-dried seaweed extract) and DPPH (% radical scavenging activity).

**Table 5:** Experimental responses obtained using the optimized ultrasound-assisted extraction conditions in brown macroalgae (*Laminaria hyperborea* and *Ascophyllum nodosum*). The results are expressed as mean ± standard deviation of the mean (*n* = 6).

Optimum Conditions	Targeted Compounds-Bioactivities ^γ^	Brown Macroalgae Species	
		*L. hyperborea*	*A. nodosum*
Condition 1	Fucose	Fucose (865.6 ± 72.9)	Fucose (2268.9 ± 178.7)
Condition 2	Total glucans	Total glucans (10,818.9 ± 22.4)	Total glucans (1127.6 ± 16.1)
Condition 3	FRAP DPPH	FRAP (10.2 ± 0.1) DPPH (33.3 ± 0.4)	FRAP (160.4 ± 5.1) DPPH (87.0 ± 1.2)
Condition 4	Fucose Total glucans FRAP DPPH	Fucose (839.0 ± 53.9) Total glucans (9530.9 ± 68.2) FRAP (11.0 ± 0.1) DPPH (44.2 ± 1.1)	Fucose (1169.3 ± 150.6) Total glucans (766.3 ± 11.8) FRAP (104.3 ± 2.4) DPPH (85.7 ± 0.7)

^γ^ The units of the experimental responses are expressed as follows: fucose (mg/100 g dried seaweed), total glucans (mg/100 g dried seaweed), FRAP (µM trolox/mg freeze-dried seaweed extract) and DPPH (% radical scavenging activity).

**Table 6:** Independent variables and coded values used for ultrasound-assisted extraction optimization.

Independent Variables	Symbols	Coded Levels		
		−1	0	+1
Temperature (°C)	X1	40	60	80
Time (min)	X2	10	20	30
Amplitude (%)	X3	40	70	100
